# Open-label clinical trial of bezafibrate treatment in patients with fatty acid oxidation disorders in Japan; 2nd report QOL survey

**DOI:** 10.1016/j.ymgmr.2019.100496

**Published:** 2019-07-25

**Authors:** Hideaki Shiraishi, Kenji Yamada, Eishin Oki, Mika Ishige, Toshiyuki Fukao, Yusuke Hamada, Norio Sakai, Fumihiro Ochi, Asami Watanabe, Sanae Kawakami, Kazuyo Kuzume, Kenji Watanabe, Koji Sameshima, Kiyotaka Nakamagoe, Akira Tamaoka, Naoko Asahina, Saki Yokoshiki, Takashi Miyakoshi, Koji Oba, Toshiyuki Isoe, Hiroshi Hayashi, Seiji Yamaguchi, Norihiro Sato

**Affiliations:** a Department of Pediatrics, Hokkaido University School of Medicine, North 15, West 7, Kita-ku, Sapporo 060-8638, Japan; b Department of Pediatrics, Shimane University Faculty of Medicine, 89-1, En-ya-cho, Izumo, Shimane 693-8501, Japan; c Department of Pediatrics, Tsugaru General Hospital, 12-3, Iwaki-cho, Goshogawara, Aomori 037-0074, Japan; d Department of Pediatrics and Child Health, Nihon University School of Medicine, 1-6, Kanda-Surugadai, Chiyoda-ku, Tokyo 101-8309, Japan; e Department of Pediatrics, Graduate School of Medicine, Gifu University, 1-1, Yanagido, Gifu 501-1194, Japan; f Child Healthcare and Genetic Science Laboratory, Division of Health Sciences, Osaka University Graduate School of Medicine, 1-7 Yamada-oka, Suita, Osaka 565-0871, Japan; g Department of Pediatrics, Osaka Hospital, Japan Community Healthcare Organization, 4-2-78, Fukushima, Fukushima-ku, Osaka 553-0003, Japan; h Department of Pediatrics, Yawatahama City General Hospital, 638, Ohira-ichibankochi, Yawatahama, Ehime 796-8502, Japan; i Department of Pediatrics, Ehime University Graduate School of Medicine, Shitsukawa, Toon, Ehime 791-0295, Japan; j Department of Community and Emergency Medicine, Ehime University School of Medicine, Shitsukawa, Toon, Ehime 791-0295, Japan; k Department of Pediatrics, Kagoshima City Hospital, 37-1, Uearata-cho, Kagoshima 890-8760, Japan; l Department of Neurology, Division of Clinical Medicine, Faculty of Medicine, University of Tsukuba, 1-1-1, Tennoudai, Tsukuba, Ibaraki 305-8575, Japan; m Hokkaido University Hospital Clinical Research and Medical Innovation Center, Research and Development Division, North 14, West 5, Kita-ku, Sapporo 060-8648, Japan; n Department of Biostatistics, School of Public Health, Graduate School of Medicine, The University of Tokyo, 7-3-1, Hongo, Bunkyo-ku, Tokyo 113-0033, Japan; o Hokkaido University Hospital Clinical Research and Medical Innovation Center, North 14, West 5, Kita-ku, Sapporo 060-8648, Japan

**Keywords:** Bezafibrate, Fatty acid oxidation disorders (FAODs), Very long-chain acyl-CoA dehydrogenase (VLCAD) deficiency, Carnitine palmitoyltransferase-II (CPT-2) deficiency, Clinical trial, Quality of life

## Abstract

**Introduction:**

Fatty acid oxidation disorders (FAODs) are rare diseases caused by a defective mitochondrial fatty acid oxidation (FAO) enzyme. We recently reported that bezafibrate improved patient quality of life (QOL) based on the SF-36 questionnaire score in patients with FAODs during a 50-week, open-label, clinical trial. Herein we conducted further survey assessments of the trial patients to define the long-term efficacy and safety of bezafibrate.

**Materials and methods:**

This trial was an open-label, non-randomized, and multicenter study of bezafibrate treatment in five patients with very long-chain acyl-CoA dehydrogenase (VLCAD) deficiency and one patient with carnitine palmitoyltransferase-II (CPT-2) deficiency (median age, 15.9 years; range, 5.8–26.4 years). The bezafibrate administration was continued for a further 102–174 weeks after the 24-week treatment described in our previous study. QOL was quantitated using the 36-Item Short Form Health Survey (SF-36) questionnaire, which constitutes eight components: physical functioning (PF), role limitation due to physical problems, bodily pain, general health perception, vitality, social functioning, role limitation due to emotional problems, and mental health.

**Results:**

PF was elevated in all patients and continued to rise during the study, with the total QOL scores increased from baseline in five of the six cases. In particular, three patients older than 20 years showed treatment efficacy, and all subcategories of QOL were elevated in two of these cases.

**Conclusion:**

Our findings supported one of the stated benefits of bezafibrate in improving QOL for patients with FAODs.

## 1 Introduction

Bezafibrate [2-(p-(2-(p-chlorobenzamido)ethyl)-phenoxy)-2-methyl propionic acid] is a peroxisome proliferator-activated receptor agonist [1] that decreases serum lipid levels in humans [2,3]. Recent reports implicated bezafibrate as a promising drug for fatty acid oxidation disorders (FAODs) based on enhanced transcription of several β-oxidation enzymes *in vitro* [[4], [5], [6], [7], [8], [9], [10]]. We also recently reported on the efficacy of bezafibrate for FAODs in a recent clinical trial [11], wherein patients showed improvements in some quality of life (QOL) components during a 6-month survey, but no significant changes in the frequency of myopathic attacks, the levels of creatine kinase (CK) and acylcarnitines (ACs, C14:1 or C16 + C18:1), and visual analog scale (VAS) values during the attacks. We suspected that these endpoints could not be adequately evaluated due to several limitations, including the small trial population. We did however find that QOL showed improvement during the 50-week study. Herein we report on a follow-up study of the trial for more than two years to evaluate the efficacy of bezafibrate for QOL in patients with FAODs.

## 2 Materials and methods

Our current study is a follow-up of previously reported results [11], using the same subjects and study design.

### 2.1 Design

This study was a non-randomized, uncontrolled, multicenter, open-label trial.

### 2.2 Standard protocol approvals, registrations, and patient consents

The Institutional Review Boards of Hokkaido University Hospital and the other collaborating institutions approved the recent clinical trial. All patients were registered on the database of the Department of Pediatrics, Shimane University Faculty of Medicine. Written informed consent for study participation was obtained from all patients or their parents.

### 2.3 Setting

The study was conducted at Hokkaido University Hospital Clinical Research and Medical Innovation Center, Japan, and the bezafibrate clinical trial was performed at each collaborating institution. Participants were recruited from January 2014 to December 2015. Bezafibrate was purchased from Kissei Pharmaceutical Co., Ltd., Nagano, Japan.

### 2.4 Patients

According to our previous study, we followed up five patients with very long-chain acyl-CoA dehydrogenase deficiency (VLCADD) and a patient with carnitine palmitoyltransferase-II deficiency (CPT2D). Patient profiles are listed in Table 1, with VL-4 and VL-5 reported in detail in previous manuscripts [12,13].

**Table 1:** Clinical features and genotypes of the patients before enrollment.

	VL-1	VL-2	VL-3	VL-4	VL-5	CP-1
Age	26 y	25 y	6 y	6 y	22 y	5 y
Sex	F	M	F	M	F	F
Diagnosis	VLCADD	VLCADD	VLCADD	VLCADD	VLCADD	CPT-2D
Industry dose of bezafibrate	400 mg/day	400 mg/day	100 mg/day	100 mg/day	400 mg/day	100 mg/day
Standard dose of bezafibrate	600 mg/day	600 mg/day	200 mg/day	200 mg/day	600 mg/day	200 → 300 mg/day
Mutation	F113^⁎^	Untested	R229X	L243F	E285G	F383Y
	K382Q	Untested	K382Q	V547 M	V400 M	R477W
Onset age	1.5 y	5 mo	Around 1 y	3 y	Around 13 y	3.7 y
Diagnosis age	5 y	13 y	0 mo	3 y	22 y	8 mo
Body weight (kg)	56	58	20	21	47	17
Clinical features	Myalgia or fatigue	Myalgia or fatigue	Myalgia or fatigue	Myalgia or rhabdomyolysis	Myalgia or rhabdomyolysis	Rhabdomyolysis or hyper CK

Frequency of						
Severe attacks	20 /year	0	Several times /year	1–2/year	5/year	1/year
Moderate attacks	50–60 /year	0	12 /y	0	7 /year	0
Mild attacks	Almost every day	2/year	Uncountable	0	12 /year	0

Treatments						
Carnitine (mg/day)	750 mg	400–600 mg	Almost none	None	1800 mg	900–1800 mg
MCT oil/milk	None	Yes	None	Yes	None	None
Restriction of activity	Prolonged walk and standing	Airing	Unclear	None	None	None
Responsiveness of bezafibrate in vitro	Good	Untested	Good	Good	Good	Untested
CK baseline (IU/L)	1933 ± 1220	768 ± 612	1112 ± 1253	81 ± 13	590 ± 660	308 ± 169
C14:1 baseline (μM)	10.41 ± 4.64	3.27 ± 4.05	2.98 ± 0.88	1.37 ± 1.77	1.36 ± 0.85	
C16 + C18:1 baseline (μM)						6.94 ± 5.70

y, year; mo, month; M, male; F, female; VLCADD, very long-chain acyl-CoA dehydrogenase deficiency; CPT-2D, carnitine palmitoyltransferase-2 deficiency; PE, physical education. Frequency of attacks and treatments were provided as of the last year before enrolment. Responsiveness to bezafibrate in vitro was evaluated using a probe acylcarnitine assay [8].

Age brackets were assigned, with three patients aged 3–7.5 years, none aged 7.5–12 years, and the remaining three aged >12 years.

### 2.5 Protocol

**Fig. 1:**
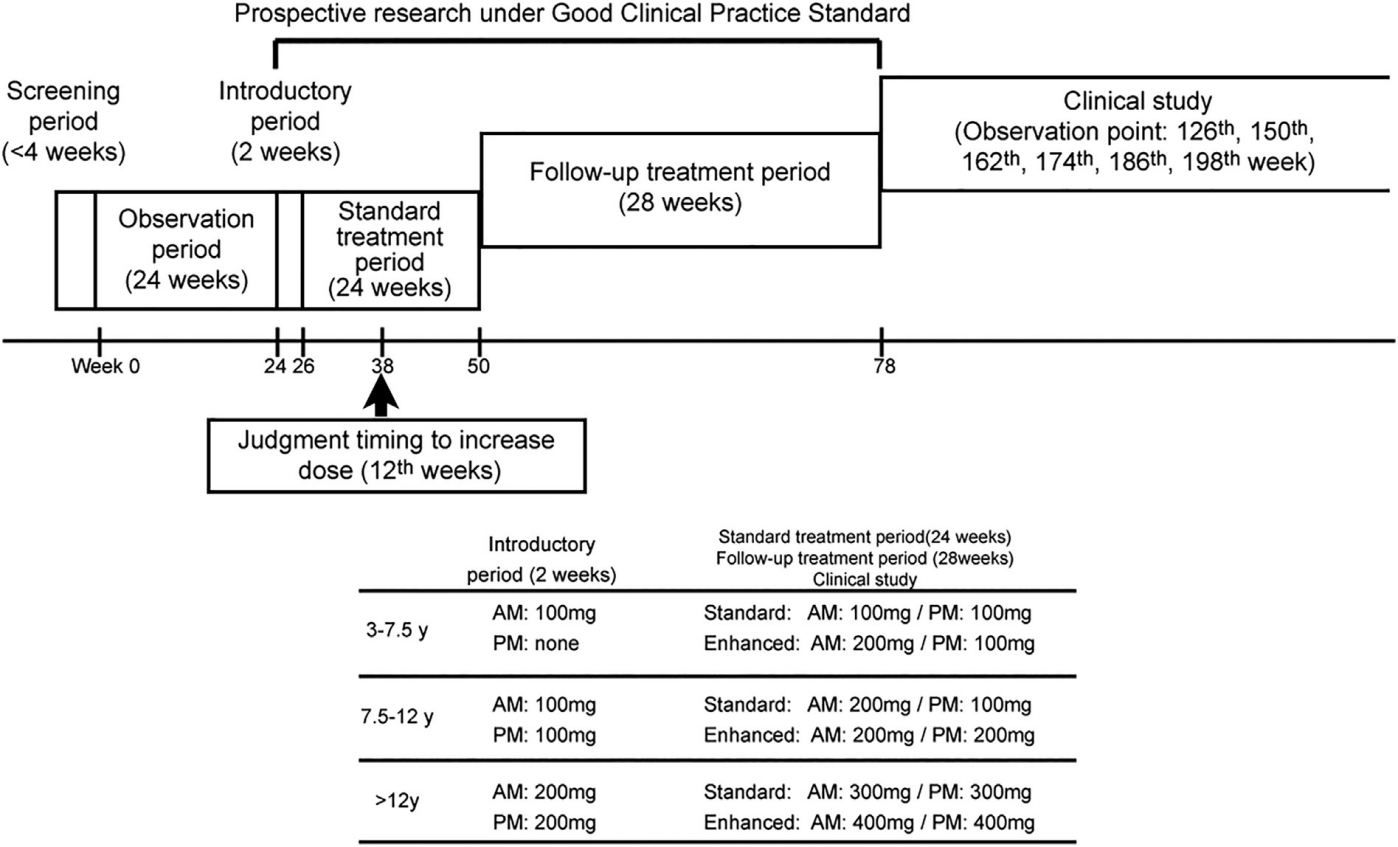
Study protocol. CP-1 patient was classified as having a severe clinical disease course at the 38th week (12 weeks after starting standard treatment) and was given enhanced-dose bezafibrate.

As shown in Fig. 1, the last study comprised 3 periods: 1) a screening period for 4 weeks; 2) an observation period for 24 weeks; and, 3) an administration period for 26 weeks. The administration period was divided into a 2-week introductory period and a 24-week standard treatment period. After the standard treatment period, all patients were invited for follow-up treatment over 28 weeks to evaluate the long-term safety of bezafibrate. The administration and follow-up treatment were conducted as prospective research under the guidance of the Good Clinical Practice (GCP) standard overseen by the Health, Labor and Welfare Ministry of Japan. After the prospective research, six of eight patients proceeded to the clinical study period, wherein QOL was evaluated using a 36-item Short Form Health Survey (SF-36) [14] to comprise this current study. The remaining two patients dropped out of the original study cohort due to economic reasons.

Table 1 details the bezafibrate dosage for each patient. Treatment with standard-dose bezafibrate (200, 300, and 600 mg/day for patients aged 3–7.5, 7.5–12, and > 12 years, respectively) was initiated and continued for 24 weeks. Enhanced treatment of 400 mg/day was accepted in one patient (CP-1) following no decrease in the frequency of myopathic attacks by the 12th week of treatment. Patient QOL was re-evaluated at the end of the administration period using the SF-36, and then again at weeks 126 (VL-4), 150 (VL-5), 162 (CP-1), 174 (VL-3), 186 (VL-2), and 198 (VL-1). The different evaluation duration for each patient reflected the initiation time of bezafibrate; however, each patient completed the questionnaire at equivalent time points. Drug compliance was calculated as (prescription number – remainder number)/prescription number.

### 2.6 Endpoint

We quantitated QOL using the SF-36 questionnaire to capture the eight components of physical functioning (PF), role limitation due to physical problems (role physical; RP), bodily pain (BP), general health perception (GHP), vitality (VT), social functioning (SF), role limitation due to emotional problems (role emotional; RE), and mental health (MH). An elevated total score indicated improvement in QOL. We have precisely surveyed any adverse effect and assessed the toxicity of bezafibrate.

### 2.7 Statistical analysis

QOL data were analyzed using a paired *t*-test for comparisons using JMP version 14.3.0 (SAS Institute Inc., Cary, NC, USA). Descriptive statistics (mean ± standard deviation) were used when repeated measurements were obtained. We defined *P* values <.05 as signifying statistical significant differences.

## 3 Results

### 3.1 QOL using the SF-36 questionnaire

**Fig. 2:**
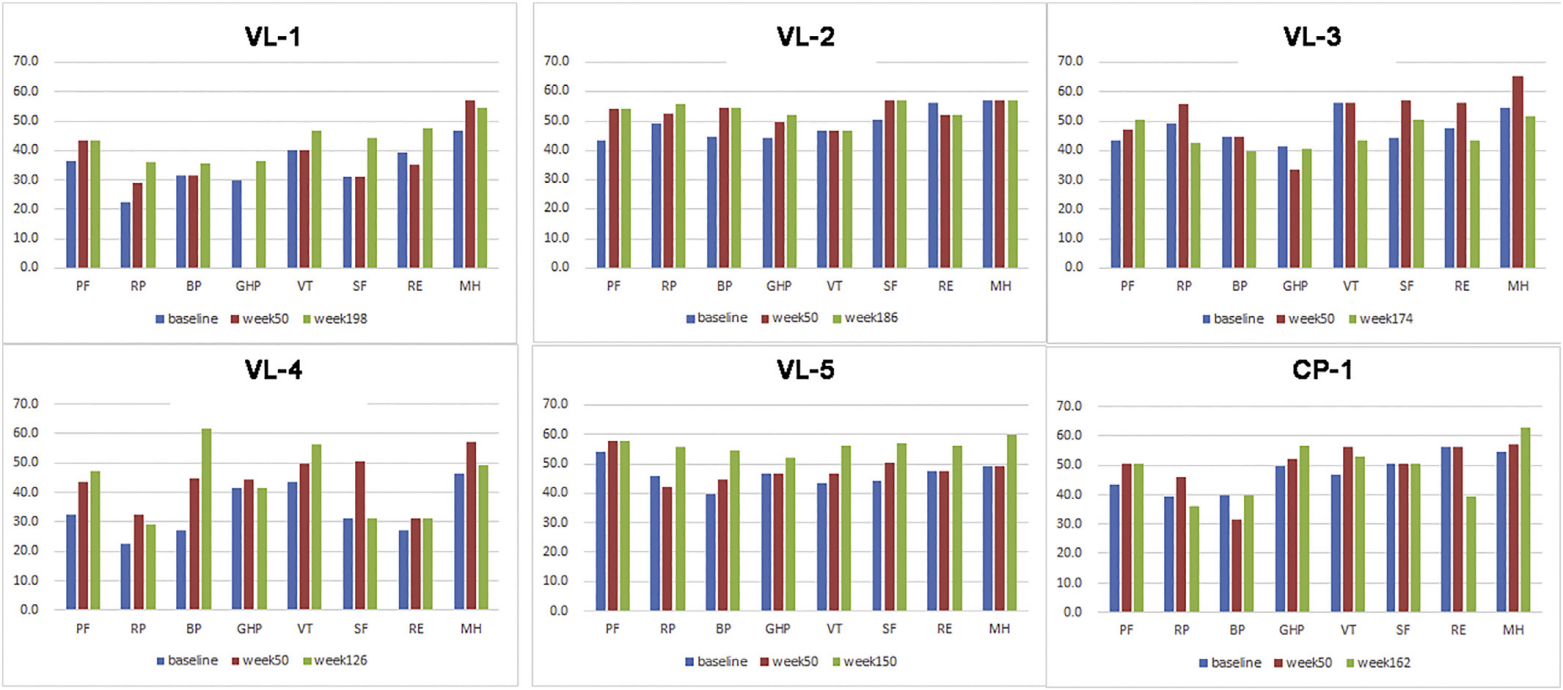
Quality of life evaluation in each patient. Color bars indicate the clinical value: SF-36 scores at baseline, week 50, and at the last survey point, respectively. PF, physical functioning; RP, role limitation due to physical problems; BP, bodily pain; GHP, general health perception; VT, vitality; SF, social functioning; RE, role limitation due to emotional problems; MH, mental health.

Results of the QOL questionnaire were obtained after the screening period (baseline), after week 50 (after standard administration), and during the clinical study (long-term evaluation) (Fig. 2).

In our previous 50-week study, the average total QOL score was significantly elevated after the administration period compared to baseline [11]. Herein we similarly found elevated QOL scores from baseline in all cases except VL-3, while the total QOL score over the extended evaluation time was further elevated compared with those at week 50 in VL-1, −2, and − 5 (Table 2). Importantly, the total QOL scores elevated steadily in all patients aged over 20 years: VL-1, VL-2, and VL-5, while showing no significant elevations among cases younger than 20 years (Table 2).

**Table 2:** Comparison of the total QOL scores using SF-36 before and after treatment.

	Age	Baseline	Week 50	Clinical study
Over 20 years old				
VL-1	26y	247.4	267.8	307.5 (198th week)
VL-2	25y	392	423.4	429.4 (186th week)
VL-5	22y	371	385.8	449.6 (150th week)
Average		336.8	359 (*P* = .045 vs. baseline)	395.5 (*P* = .038 vs. baseline) (*P* = .161 vs. week 50)

Under 20 years old				
VL-3	6y	381.3	415.5	362.7 (174th week)
VL-4	6y	271.5	353.5	347.0 (126th week)
CP-1	5y	379.7	400.2	388.2 (162nd week)
Average		344.2	389.7 (*P* = .134 vs. baseline)	366.0 (*P* = .517 vs. baseline) (*P* = .245 vs. week 50)
Total Average		340.5	374.4 (*P* = .020 vs. baseline)	380.7 (*P* = .053 vs. baseline; *P* = .719 vs. week 50)

Elevation of the QOL score indicates improvement in QOL among total cases, and cases older or younger than 20 years old.

This survey revealed maintenance of the improvement over a long-term evaluation for all QOL components with the exception of RE (Table 3); however, the results of this clinical study were not significantly improved compared with those over 50 weeks.

**Table 3:** Averaged QOL results from the SF-36 administered before and after treatment.

	Baseline	Week 50 (*P-*value vs. baseline)	Clinical study (*P-*value vs. baseline)	*P-*value: week 50 vs. clinical study
Physical functioning (*n* = 6)	42.2	49.4 (*P* ≤ .01)	50.6 (*P* = .003)	0.175
Physical role (*n* = 6)	38.0	43.0 (*P* = .019)	42.4 (*P* = .224)	0.897
Bodily pain (*n* = 6)	39.7	41.9 (*P* = .109)	47.7 (*P* = .151)	0.130
General health perceptions (*n* = 5)	42.2	45.3 (*P* = .354)	46.5 (*P* = .041)	0.117
Vitality (*n* = 6)	46.1	49.3 (*P* = .194)	50.4 (*P* = .329)	0.767
Social functioning (*n* = 6)	42.0	49.5 (*P* = .033)	48.4 (*P* = .041)	0.821
Emotional role (*n* = 6)	45.7	46.4 (*P* = .315)	45.0 (*P* = .869)	0.775
Mental health (*n* = 6)	51.4	57.2 (*P* = .070)	55.9 (*P* = .094)	0.726

### 3.2 Safety and toxicity of bezafibrate

A patient took herpangina during the 24-week observation period prior to the administration period. This issue does not affect the current results since bezafibrate was not used. Other patients kept using bezafibrate without any adverse effect or toxicity to liver and kidney function. Levels of total cholesterol remained within normal limits.

## 4 Discussion

A French group undertook the first study of bezafibrate treatment for FAODs [9]. Based on that work, we undertook the next prospective clinical study in compliance with GCP standards and as officially approved by the Health, Labor and Welfare Ministry of Japan [5,11]. Our findings revealed a short-term efficacy for bezafibrate in patients with FAODs and suggested that the subjective QOL of patients was improved during the 50-week study. Herein, we report a longer-term, follow-up study wherein we continued to administer bezafibrate after the initial 50-week period (completion of the previous trial) and continued monitoring of QOL using the SF-36 questionnaire. In five of the six cases, QOL improved by >50% across all quality sub-components. Two patients showed QOL improvement in all sub-components, while all cases aged over 20 years including a 25-year-old showed remarkable QOL improvement. In particular, one patient assessed as a non-responder to bezafibrate in the original study showed a remarkable improvement in QOL score during this follow-up study. Specifically, her number of hospitalizations decreased, and she showed a response to bezafibrate, suggesting that long-term administration is needed to achieve a benefit for some patients with FAODs. Our results also suggested that the effect of bezafibrate on QOL continued for at least 2 years.

In contrast, cases under the age of 20 years, and particularly those under 10 years, showed a decreased score for some QOL sub-components, despite their caregiver reporting that daily activity and exercise increased accordingly in these patients. The decrease in some aspects of QOL in the younger participants might therefore reflect their increased general activity even if bezafibrate suppressed the metabolic attacks. In addition, no cases refused to continue taking bezafibrate, suggesting that participant and/or their caregivers recognized the efficacy of bezafibrate. In general, an effective drug for movement disorder or neuropraxia should gain activity for the patient. Thus, an appropriate and objective procedure should be established to assess the efficacy of new drugs for FAOD. For example, objective assessment of daily activity including length of walking ability and duration of exercise might be a suitable method for assessing the efficacy of some drugs for patients with FAOD.

Our previous study found no improvement in CPK level during myopathic attack, Visual Analog Scale scores, or acylcarnitine concentrations from bezafibrate treatment in this population. One problem with the previous manuscript was the definition of myopathic attack as when patients show a 5-fold increase over baseline CK levels; however, the levels tended to increase gradually for 4 days, so complaint of myopathic attack could not be confirmed, even when the patients complained of general fatigue or myalgia, and visited the hospital for treatment. So, we should ignore real myopathic attack and the assessment of AC should not be promising. In this way, the primary endpoint of our previous study was not defined statistically.

To adequately assess the efficacy of a new drug for FAOD, we therefore need a new technique that demonstrates changes in mitochondrial function directly. Bastin et al. tried to assess the efficacy of drugs based on myoblast cell activity from a biopsy collected before and after administration of bezafibrate; however, this procedure would be too invasive for enrolled patients. Another possible new technique is direct observation of mitochondria using immunoluminescence analysis. Murchison et al. demonstrated that voltage-clamp electrophysiology with Ca2^+^-sensitive ratiometric microfluorimetry and laser scanning confocal microscopy showed the participation in Ca2^+^ buffering of in situ mitochondria in a model rat with mitochondrial dysfunction [15]. A potential-dependent, J-aggregate-forming, delocalized lipophilic cation, 5,5′,6,6′-tetrachloro- 1,1′,3,3′-tetraethylbenzimidazolocarbocyanine iodide (JC-1), is another promising procedure that shows membrane potentials across mitochondria in a living cell [16]. Although these techniques require a fibroblast biopsy for subjective patients, they should still be tested to assess mitochondrial function.

There remains no effective cure for FAODs, and adequate ingestion of carbohydrates with administration of MCT milk is regarded as the only treatment [17]. An earlier clinical trial showed the in vitro efficacy of bezafibrate to treat FAODs, and we believe that the present findings will further help patients adapt to bezafibrate therapy [[4], [5], [6], [7], [8], [9], [10]]. In addition, no adverse events have been recorded for bezafibrate thus far. Finally, we could not fully discount the possibility of placebo effect in this study, since we evaluated subjective QOL using an open-label survey.

## 5 Conclusion

Our study indicated that bezafibrate treatment might be effective in improving QOL for patients with FAODs for over 2 years without adverse effects.

## Contributions

H. Shiraishi and K. Yamada contributed equally to the submission of this manuscript by participating in the study conception and design, figure acquisition, and data interpretation, and by drafting, revising, and finalizing the manuscript. Additionally, H. Shiraishi, S. Yamaguchi, and N. Sato extensively organized the writing of this manuscript and performed and designed this trial as principal investigators. N. Asahina first conceived this trial and participated in the study by acquiring funding, analyzing and interpreting the data, and revising the manuscript. S. Yokoshiki, T. Miyakoshi, T. Isoe, and H. Hayashi contributed to the registration, design, governance, proceeding, and intermediacy of this trial as the main clerical organizers of this study. The trial statistician, K. Oba, participated in the study conception and design and the data analysis and interpretation. E. Oki, M. Ishige, T. Fukao, Y. Hamada, N. Sakai, F. Ochi, A. Watanabe, S. Kawakami, K. Kuzume, K. Watanabe, K. Sameshima, K. Nakamagoe, and A. Tamaoka contributed to the design of this trial and provided clinical data, suggestions, and data interpretation as attending doctors and investigators.

## Sources of funding

This study was supported by “The multicenter clinical trial of the efficacy and safety of bezafibrate for mitochondrial fatty acid β-oxidation disorders (Grant Number, 16lk0103005h0005)” from the Japan Agency for Medical Research and Development (AMED).

## Declaration of Competing Interest

None of the authors have conflicts of interest to declare regarding the publication of this manuscript.
